# A Case of Painful Legs and Moving Toes Syndrome Mimicking Restless Legs Syndrome

**DOI:** 10.7759/cureus.109117

**Published:** 2026-05-18

**Authors:** Yu Li, Risa Maeda, Yoshihide Okano, Tetsuya Kanai, Takanori Uehara

**Affiliations:** 1 General Medicine, Chiba University Hospital, Chiba, JPN; 2 Internal Medicine and Neurology, Kanai Medical Clinic, Kamatori, JPN

**Keywords:** chronic foot pain, involuntary movements, painful legs and moving toes syndrome, plmt, rare neurological disorders, restless legs syndrome, rls

## Abstract

Painful legs and moving toes syndrome (PLMT) is a neurological disorder characterized by leg pain accompanied by involuntary toe movements. It may be misdiagnosed as restless legs syndrome (RLS) due to overlapping clinical features. We report the case of a 69-year-old woman with a seven-year history of pain involving all toes of both feet that improved with standing or walking and worsened when lying down. She had no sleep disturbance and was unaware of involuntary movements. A trial of dopamine agonist therapy was ineffective. Careful observation revealed involuntary toe movements, supporting a clinical diagnosis of PLMT. Neuroimaging revealed lumbosacral lesions, but no findings clearly explained her symptoms, and nerve conduction studies showed no evidence of peripheral neuropathy. This case highlights the importance of observing the patient’s feet over a prolonged period and considering PLMT in patients with chronic leg pain that is exacerbated at rest but lacks typical RLS features.

## Introduction

Painful legs and moving toes syndrome (PLMT), first described by Spillane et al. [1], is a neurological condition characterized by persistent leg pain accompanied by involuntary toe movements [2]. In some cases of PLMT, pain may improve with weight bearing, such as standing or walking. Restless legs syndrome (RLS), classically described by Ekbom, is characterized by an urge to move the legs, typically accompanied by uncomfortable, sometimes painful sensations that worsen at rest and improve with movement [3]. Both conditions may lead to significant distress and reduced quality of life. This similarity in relieving factors can lead to misdiagnosis. Here, we report a case of PLMT in which pain improved with standing or walking. It was initially suspected to be RLS but was ultimately diagnosed as PLMT.

## Case presentation

A 69-year-old woman presented with a seven-year history of toe pain that had progressed to involve the front half of the soles. Her medical history includes lung cancer surgery two years earlier and a history of depression 16 years prior; however, there had been no recurrence of lung cancer on regular follow-up with PET-CT, and her depression was stable without current symptoms such as depressed mood, reduced interest, or fatigue. She had no history of trauma to the legs. The pain was persistent, improved with standing or walking, and worsened when lying down. It also worsened in the evening and at night, although there were no sleep disturbances. She denied any awareness of involuntary movement. She had previously been evaluated by orthopedics, neurology, and vascular specialists. Nerve conduction studies, brain MRI, lumbar spine MRI, and vascular ultrasonography did not identify a cause of her symptoms. Neurological examination revealed normal muscle strength, including ankle dorsiflexion and plantarflexion, with normal muscle tone and no rigidity. Deep tendon reflexes were normal and symmetric, and Babinski signs were absent bilaterally. Sensory examination in the lower extremities, including light touch, pain and temperature sensation, and vibration sensation, was normal. Coordination was intact, with a negative Romberg test, preserved single-leg standing bilaterally, and normal gait. Symptomatic treatment with mirogabalin was prescribed, but its effect was limited. Her exacerbating and relieving factors, including worsening from evening to night and improvement with standing or walking, reminded us of RLS, but the absence of sleep disturbance was not typical for RLS. A two-week trial of pramipexole, a dopamine agonist, was undertaken to evaluate the possibility of RLS, but the response was poor. Careful observation of her toes while talking with her revealed slow, irregular, nonrhythmic movements involving all toes of both feet, including flexion, extension, adduction, and abduction (Videos 1, 2).

**Video 1:** Involuntary movement of the toes when sitting

**Video 2:** Involuntary movement of the toes when lying

The patient was unaware of these movements. A neurologist also evaluated the involuntary movements of her toes, and PLMT was clinically suspected. To rule out the secondary cause of PLMT, we reviewed the results of brain MRI, lumbar spine MRI, and nerve conduction studies. Brain MRI showed no structural abnormalities, including stroke, tumor, or demyelinating disease. Lumbar spine MRI revealed no significant spinal canal stenosis, foraminal stenosis, or nerve root compression. A 7-mm intradural extramedullary lesion at the L2/3 vertebral level, suggestive of a schwannoma, and a perineural cyst at the S2 vertebral level were also identified within the cauda equina. However, neither lesion was considered likely to account for the patient’s symptoms. This is because her pain and involuntary movements were bilateral, predominantly distal, centered on the toes, and did not correspond to a single lumbosacral nerve root distribution. In addition, she had no sacral or perineal pain, sensory disturbance, or bladder or bowel dysfunction that would suggest symptomatic cauda equina syndrome. In evaluating the observed involuntary movements, spinal segmental myoclonus was also considered as a differential diagnosis. However, it was considered unlikely because the lesions were located within the cauda equina rather than the spinal cord, and the movements were neither sustained nor rhythmic and lacked shock-like features, which are not typical of spinal segmental myoclonus. Nerve conduction studies showed no evidence of peripheral neuropathy. No additional investigations identified abnormalities that could explain her symptoms.

## Discussion

PLMT is characterized by leg pain accompanied by involuntary movement of the toes [2]. Involuntary movements reported in PLMT include abduction/adduction, fanning, and clawing of the toes in addition to flexion/extension [2]. These diverse and complex movement patterns are difficult to reproduce voluntarily and may help support the involuntary nature of the movements in this syndrome. These involuntary movements typically accompany pain at onset but may also appear months or even years later [2]. Moreover, patients may be unaware of them, and the movements may not be constantly present, as in the present case. This may require prolonged observation of the toes. Most cases of PLMT are idiopathic (42%) but are sometimes secondary due to peripheral neuropathy (28%), trauma (11%), or radiculopathy (9%) [2]. The pathophysiologic mechanism remains unclear. Pain exacerbating or alleviating features are often identified (58%), including various positions (sitting, walking/weight bearing, or bending) [2]. RLS can sometimes be a differential diagnosis of PLMT, and we propose a summary of the differences, as shown in Table 1 [2,4-6].

**Table 1:** Difference between painful legs and moving toes syndrome (PLMT) and restless legs syndrome (RLS) *Whether it worsens or improves varies among cases.

	PLMT［2,5］	RLS［4,6］
Exacerbating features	Sometimes worsens with weight bearing*. Diurnal variation can be observed.	Worsens at rest. Worsens in the evening or at night
Alleviating features	Sometimes improves with weight bearing*	Improves with movement
Sleep disturbances	Frequency unclear	Common
Involuntary movement	Often present during wakefulness to light sleep	Often present during sleep (periodic limb movements of sleep)
Dopamine agonist trial	Often limited	Often responsive, though not always first line

Although the observed clinical phenomenology was considered compatible with PLMT, the absence of detailed electrophysiologic characterization of the involuntary movements remains a limitation of this report. Additional electrophysiologic evaluation, such as needle electromyography during the movements, may have further strengthened the diagnostic distinction from other movement disorders, including spinal segmental myoclonus. In addition, electromyographic evaluation during periods without visually apparent involuntary movements might have detected abnormal muscle activity not evident on routine observation.

## Conclusions

PLMT may mimic RLS when pain characteristics overlap, and distinguishing the two conditions may be clinically challenging, particularly when involuntary movements are subtle or intermittently observed. In this case, the absence of sleep disturbance and a poor response to dopamine agonists prompted careful examination for involuntary toe movements. Prolonged observation of the feet was helpful, as the patient was unaware of the movements. PLMT may be worth considering in patients with similar presentations. Pain in PLMT is often intractable, and no clearly established treatment has been identified. Thus, further case accumulation and research are warranted.
